# Nano-Sized Graphene Oxide Attenuates Ovalbumin/Alum-Induced Skin Inflammation by Down-Regulating Th2 Immune Responses in Balb/c Mice

**DOI:** 10.3390/biom14080962

**Published:** 2024-08-07

**Authors:** Hyun Jung Park, Sung Won Lee, Luc Van Kaer, Suklyun Hong, Seokmann Hong

**Affiliations:** 1 Department of Integrative Bioscience and Biotechnology, Institute of Anticancer Medicine Development, Sejong University, Seoul 05006, Republic of Korea; 0402parkhj@gmail.com; 2 Department of Biomedical Laboratory Science, College of Health and Biomedical Services, Sangji University, Wonju 26339, Republic of Korea; sungwonlee@sangji.ac.kr; 3 Department of Pathology, Microbiology and Immunology, Vanderbilt University School of Medicine, Nashville, TN 37232, USA; luc.van.kaer@vumc.org; 4 Department of Physics, Graphene Research Institute, and GRI-TPC International Research Center, Sejong University, Seoul 05006, Republic of Korea; hong@sejong.ac.kr

**Keywords:** nano-graphene oxide, skin inflammation, ovalbumin, DO11.10 TCR transgenic mice, regulatory T cells

## Abstract

Graphene oxide (GO), a carbon-based material with oxygen-containing functional groups, can be applied in biomedicine for drug delivery, cancer therapy, and tissue regeneration. We have previously shown that nanoscale-sized graphene oxide (NGO), an oxidized graphene derivative, exhibits effective anti-inflammatory activity in a murine model of sepsis mediated by T helper (Th)1-promoting cytokines such as IFNγ and TNFα. However, whether NGO influences Th2-induced skin inflammation remains unclear. To address this issue, we employed an ovalbumin (OVA) plus aluminum hydroxide (Alum)-induced Th2-mediated skin inflammation model in conjunction with OVA-specific DO11.10 T cell receptor transgenic Balb/c mice. In vivo NGO injection upon OVA/Alum sensitization down-regulated OVA-elicited antigen-specific Th2 cells and GATA3-expressing Th2-type regulatory T cells. Next, we examined the effect of NGO injection on OVA/Alum-induced atopic dermatitis (AD)-like skin inflammation. NGO-injected mice exhibited significantly decreased Th2 disease phenotypes (e.g., a lower clinical score, decreased epidermal thickness and Th2 cell differentiation, and fewer infiltrated mast cells and basophils in skin lesions) compared with vehicle-injected control mice. Overall, our results suggest that NGOs are promising therapeutic materials for treating allergic diseases such as AD.

## 1. Introduction

Atopic dermatitis (AD) is a chronic cutaneous inflammatory disease that causes edema, itching, scratching, erythema, and lichenification [1]. Most patients with AD display marked infiltration of leukocytes in the skin and increased epidermal thickness [2]. A decreased T helper (Th) 1/Th2 ratio in these patients results in a shift towards a dominant Th2 response related to the development of AD [3]. Thus, balancing Th1/Th2 immune responses is critical to controlling AD development [4,5]. The pro-inflammatory cytokine IL1β induces the outcome of AD skin inflammation through qualitative changes associated with epidermal homeostasis [6]. IL1β also induces inflammatory Th2 cell differentiation, and, hence, IL1β-primed Th2 cells exacerbate AD pathogenesis [7]. The ovalbumin (OVA) antigen-induced skin murine inflammation model is a well-established AD model in which OVA sensitization and challenge induce AD-like skin lesions by eliciting dominant Th2 immune responses [8].

Regulatory T (Treg) cells are characterized by expression of the transcription factor Foxp3 (forkhead box P3), and these cells physiologically play a pivotal role in immune homeostasis by suppressing immune effector lymphocytes such as Th1, Th2, and Th17 cells [9]. The functional alteration of Treg cells and decreased Treg/Th2 ratios are associated with the severity of AD pathogenesis in a murine model [4,5,10]. Food allergy (FA)-sensitive mice display an increased frequency of GATA Binding protein 3 (GATA3)^+^ Treg cells in their mesenteric lymph nodes (MLN) after OVA sensitization [11]. Moreover, Th2-like Treg cells expressing GATA3 are strongly associated with the pathogenic progression of allergic asthma [12,13].

Graphene and graphene oxide (GO) are two-dimensional materials with unique electrochemical properties, including high electronic and thermal conductivity. When GO is intravenously (i.v.) injected into mice, it accumulates in the liver, lungs, and spleen. While a low amount of i.v. injected GO (1 mg/kg body weight) shows superior biocompatibility, i.v. injection at a high dose (10 mg/kg body weight) causes pathological changes and toxicity due to long-term accumulation in the body. Therefore, it is critical to determine an appropriate non-toxic dose for in vivo application [14,15]. These materials exhibit remarkable biological applications such as drug delivery, tissue engineering, and biosensing [16]. Small and thin GO directly suppresses the production of inflammasome-related cytokines (i.e., IL1β) by macrophages in an NLRP3-independent manner [17]. In an experimental model of sepsis, nanoscale-sized graphene oxide (NGO)-mediated anti-inflammatory responses correlate with decreased IFNγ and IL4 secretion but increased TGFβ production by glycolipid-reactive invariant natural killer T (iNKT) cells [18]. In a model of OVA-induced allergic asthma, GO exposure significantly suppresses antigen-specific Th2 responses [19]. However, the effects and regulatory mechanism of GO in OVA-induced chronic skin inflammation remain unexplored. Thus, we investigated whether NGO administration affects the pathogenesis of OVA-induced skin inflammation by employing OVA-specific DO11.10 T cell receptor (TCR) transgenic (Tg) mice. Furthermore, we examined whether NGO application can influence the activation of dendritic cells (DCs) and the differentiation of CD4^+^ T cells into Th1, Th2, and Treg cells during skin inflammation.

## 2. Materials and Methods

### 2.1. Study Design

This study was designed to determine the effect of NGO on OVA-induced chronic skin inflammation. To address this issue, OVA-specific DO11.10 TCR Tg mice were intraperitoneally (i.p.) injected with aluminum hydroxide (Alum; Thermo Fisher Scientific, Rockford, IL, USA) plus OVA and were concurrently i.v. injected with either NGO (50 μg) or a phosphate-buffered saline (PBS) control once a week, totaling three injections. One week after the last injection, splenocytes were harvested and analyzed by flow cytometry. Only male mice and 4 mice per group were used in all the experiments. Sejong University Institutional Review Board approval was obtained before the experiments (SJ-20210705, 8 February 2021).

### 2.2. Mice and Reagents

Wild-type (WT) Balb/c mice were purchased from Jung Ang Lab Animal Inc. (Seoul, Republic of Korea). The IL4/GFP reporter (4Get) Balb/c mice were kindly provided by Dr. R. Locksley (University of California at San Francisco, CA, USA). The DO11.10 OVA-specific TCR Tg mice used in this study had a Balb/c genetic background, and were obtained from Dr. Se-Ho Park (Korea University, Seoul, Republic of Korea). 4Get Balb/c mice were further crossed with DO11.10 Balb/c mice to obtain 4Get/DO11.10 Balb/c mice. All mice used in this study were maintained at Sejong University and used for experiments at 6–12 weeks of age. The mice were fed a γ-irradiated sterile diet and provided with autoclaved tap water. The mice were maintained on a 12 h light/12 h dark cycle in a temperature-controlled barrier facility with free access to food and water. Age- and sex-matched mice were used for all experiments. The animal experiments were approved by the Institutional Animal Care and Use Committee at Sejong University (protocol code (SJ-20210705), approved on 2 August 2021). OVA peptide 323–339 (OVA_323–339_; ISQAVHAAHAEINEAGR) was synthesized by Peptron Inc. (Daejeon, Republic of Korea). NGO (NANO-GO-P) was purchased from Graphene Supermarket (Calverton, New York, NY, USA) and was dispersed in distilled water as the stock solution (10 mg/mL).

### 2.3. Immunization Protocols

DO11.10 TCR Tg or 4Get/DO11.10 TCR Tg Balb/c mice were immunized i.p. with Alum (2 µg) plus OVA_323–339_ peptide (100 µg), together with the simultaneous i.v. injection of either NGO (50 µg) or a vehicle once a week, totaling three injections. One week after the last injection, these groups were sacrificed by CO_2_ inhalation for the experiments, and splenocytes were prepared from these mice. Cytokine production was determined by intracellular cytokine staining.

### 2.4. Induction of OVA-Induced Skin Inflammation

DO11.10 TCR Tg Balb/c mice were immunized three times i.p. with the OVA_323–339_ peptide (100 µg) plus Alum (2 µg) once a week. Before applying OVA-containing skin patches, mice were anesthetized and, subsequently, the dorsal skin was shaved with an electric clipper and hair removal cream. The OVA patches were prepared with a sterile gauze moistened with 100 μg OVA in PBS. The OVA patches were attached to the shaved dorsal skin for seven days (i.e., from day 21 to 28) and were changed daily.

### 2.5. Flow Cytometry

The following monoclonal antibodies (mAbs) from BD Biosciences (San Jose, CA, USA) were used: fluorescein isothiocyanate (FITC)-, phycoerythrin (PE)-Cy7-, or allophycocyanin (APC)-conjugated anti-CD3ε (clone 145-2C11); PE-conjugated anti-CD44 (clone IM7); FITC-, PE-Cy7-, or APC-conjugated anti-CD11c (clone HL3); PE-Cy7- or APC-conjugated anti-MHC II (clone M5/114.15.2); FITC-, PE-, or APC-conjugated anti-CD4 (clone RM4-5); PE-conjugated anti-GATA3 (clone L50-823). In addition, the following mAbs from Thermo Fisher Scientific (Rockford, IL, USA) were used: PE-conjugated anti-IFNγ (clone XMG1.2); PE-Cy7- or APC-conjugated anti-KJ1-26 (clone KJ1-26); PE-conjugated anti-IL1β (clone NJTEN3); PE-conjugated anti-Foxp3 (clone NRRF-30). To perform surface staining, cells were harvested and washed twice with cold 0.5% bovine serum albumin (BSA)-containing PBS (FACS buffer). To block Fc receptors, the cells were incubated with anti-CD16/CD32 mAbs on ice for 10 min and subsequently stained with fluorescently labeled mAbs. Flow cytometric data were acquired using a FACSCalibur flow cytometer (Becton Dickson, San Jose, CA, USA) and analyzed using FlowJo software (version 8.7; Tree Star, Ashland, OR, USA).

### 2.6. Intracellular Cytokine Staining

For intracellular staining, the splenocytes were incubated with brefeldin A, an intracellular protein transport inhibitor (10 μg/mL), in RPMI medium for 2 h at 37 °C. The cells were stained for cell surface markers, fixed with 1% paraformaldehyde, washed once with cold FACS buffer, and permeabilized with 0.5% saponin. The permeabilized cells were then stained for an additional 30 min at room temperature with the indicated mAbs (PE-conjugated anti-IFNγ or anti-IL1β mAb, or PE-conjugated isotype control rat IgG mAb). Fixation and permeabilization were performed using a Foxp3 staining kit (eBioscience, San Diego, CA, USA) with the indicated mAbs (PE-conjugated anti-Foxp3 or anti-GATA3 mAb, or isotype control rat IgG mAb). More than 5000 cells per sample were acquired using a FACSCalibur flow cytometer, and the data were analyzed using the FlowJo software package (Tree Star, Ashland, OR, USA).

### 2.7. Analysis of Liver and Skin Sections

Mice were anesthetized using ketamine and xylazine (40 mg/kg and 4 mg/kg, respectively) and were perfused through the left cardiac ventricle with cold PBS (pH 7.4) for 3 min to remove peripheral blood mononuclear cells (PBMCs) from the blood vessels. After perfusion, the liver and dorsal skin were prepared. Subsequently, the liver and dorsal skin were fixed in 4% paraformaldehyde, embedded in paraffin, and cut into 6 μm sections using a microtome (RM 2235, Leica, Wetzlar, Germany). The sections were then stained with hematoxylin and eosin (H&E) to analyze histological changes and stained with toluidine blue to detect mast cells. The cells were counted with a microscope at a magnification of 400 times. The cell density was expressed as the total number of cells in ten high-power fields (400×) for each section.

### 2.8. Measurement of Liver Enzyme Levels

Sera were collected from the mice, and the serum levels of the liver enzymes aspartate aminotransferase (AST) and alanine aminotransferase (ALT) were analyzed by KLSbio Co., Ltd. (Suwon, Republic of Korea).

### 2.9. Scoring the Severity of Skin Lesions

Skin lesions were scored at the indicated time points. The scoring was based on the severity of scarring/dryness, erosion/excoriation, lichenification, edema, and erythema/hemorrhage. The total clinical skin severity score was defined as the sum of the five signs (none = 0; mild = 1; moderate = 2; and severe = 3) [20].

### 2.10. Statistical Analysis

One-way ANOVA analysis was carried out using VassarStats (http://vassarstats.net/anova1u.html) (accessed on 30 July 2024). * *p* < 0.05, ** *p* < 0.01, and *** *p* < 0.001 were considered significant in the one-way ANOVA.

## 3. Results

### 3.1. NGO Suppresses Th2 Cell Differentiation Elicited by OVA/Alum Immunization

**Figure 1:**
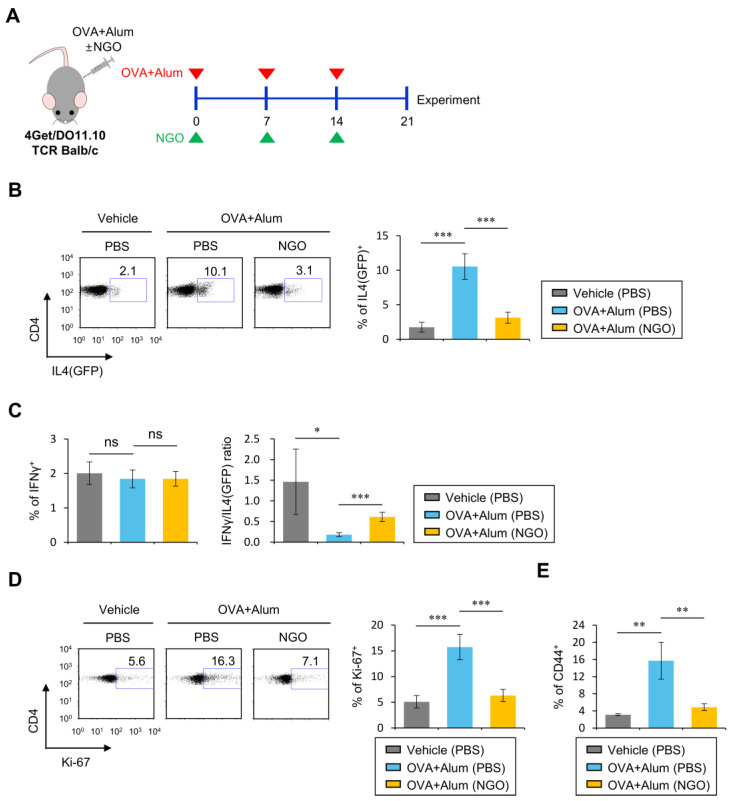
NGO injection suppresses Th2 cell differentiation after OVA/Alum immunization. (**A**) 4Get/DO11.10 TCR Tg Balb/c mice were immunized i.p. with Alum (1 mg) plus OVA_323–339_ peptide (100 µg) after i.v. injection of either NGO (50 µg) or PBS once a week for 3 weeks. One week after the last injection (at 21 days after immunization), splenocytes were prepared from both PBS- and NGO-treated mice. (**B**,**C**) The frequency of IL4(GFP)- (**B**) and IFNγ-expressing (**C**) populations in splenic KJ1-26^+^CD4^+^ T cells from each group was determined by flow cytometry. (**B**) (left) Representative FACS plots; (right) summary figure. (**D**) The expression of Ki-67 on splenic KJ1-26^+^CD4^+^ T cells was evaluated by flow cytometry. (left) Representative FACS plots; (right) summary figure. (**E**) The expression of CD44 on splenic KJ1-26^+^CD4^+^ T cells was evaluated by flow cytometry. The mean values ± SD (*n* = 4 per group in the experiment; one-way ANOVA; * *p* < 0.05, ** *p* < 0.01, *** *p* < 0.001). One representative experiment of two experiments is shown. ns, not significant.

Inflammasome-associated cytokines such as IL1β play a crucial role in initiating Th2 immune responses in AD [6,7]. We have previously shown that NGO administration significantly down-regulates inflammatory responses mediated by the iNKT cell ligand α-galactosylceramide (α-GalCer) [18]. However, it is unclear whether NGO injection can affect the immune response induced by the Th2-promoting adjuvant Alum (aluminum sulfate). To address this issue, DO11.10 TCR Tg mice were i.p. immunized with OVA/Alum and i.v. co-injected with either NGO or as PBS vehicle control (Figure 1A) three times at one-week intervals. One week after the last injection, we analyzed the cell number of and IL1β production by DCs in the spleen. While OVA/Alum injection increased the cell number of DCs and induced DCs to secrete pro-inflammatory IL1β, NGO injection significantly attenuated the generation of such pro-inflammatory DCs (Figure S1). Thus, these findings underscore the potential of NGOs in regulating the Alum-triggered activation of DCs, although the underlying mechanism is yet to be elucidated.

We have previously shown that NGO injection significantly suppresses α-GalCer-mediated antigen-specific CD4 T cell differentiation [18]. However, it is unclear whether NGO injection can affect Alum-elicited antigen-specific T cell responses. To address this issue, we immunized 4Get/DO11.10 TCR Tg Balb/c mice with OVA/Alum after the injection of either NGO or PBS and further examined Th1/Th2 cytokine profiles of CD4^+^ helper T cells from these mice via flow cytometric analysis. Interestingly, upon OVA-induced skin inflammation, we found that NGO-injected mice displayed reduced levels of IL4-producing but not IFNγ-producing populations among splenic CD4^+^ T cells expressing the clonotypic OVA-specific KJ1-26 TCR compared with PBS-injected controls, indicating that NGO treatment induces a significant reduction in Th2 cells, consequently increasing Th1/Th2 ratios (Figure 1B,C). Next, to examine whether these alterations in Th responses were associated with phenotypic changes in antigen-specific CD4^+^ T cells, we measured the expression of a proliferation marker (Ki-67) and an activation/memory marker (CD44) among KJ1-26^+^CD4^+^ T cells. We found that the Ki-67- and CD44-expressing cell frequencies of KJ1-26^+^CD4^+^ T cells in the spleen were dramatically lower in NGO-injected mice compared to the PBS-injected controls (Figure 1D,E). These results demonstrate that NGO can attenuate Alum-elicited Th2 cell proliferation and differentiation into an effector memory phenotype.

### 3.2. NGO Suppresses Th2-Type Treg Cell Differentiation after OVA/Alum Immunization

**Figure 2:**
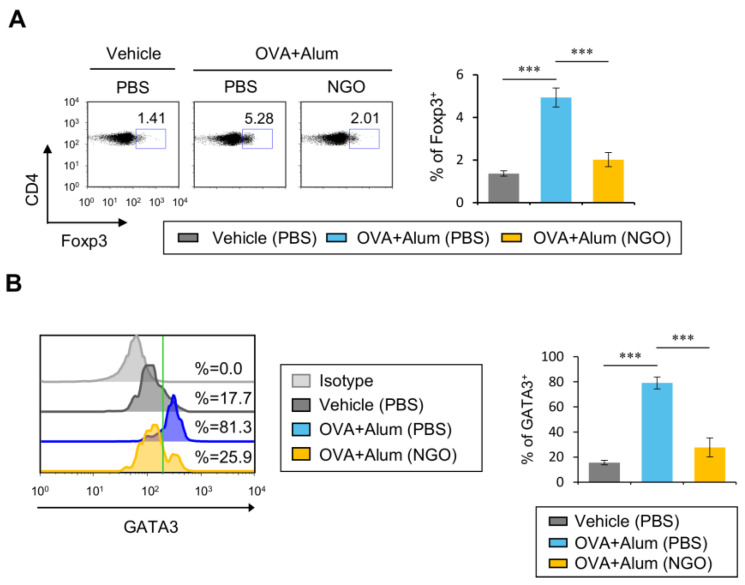
NGO injection suppresses Th2-type Treg cell differentiation after OVA/Alum immunization. (**A**,**B**) DO11.10 TCR Tg Balb/c mice were immunized i.p. with Alum (1 mg) plus OVA_323–339_ peptide (100 µg) after i.v. injection of either NGO (50 µg) or PBS once a week for 3 weeks. One week after the last injection (at 21 days after immunization), splenocytes were prepared from both PBS- or NGO-treated mice. (**A**) The intracellular expression of Foxp3 in splenic KJ1-26^+^CD4^+^ T cells from each group was analyzed by flow cytometry. (left) Representative FACS plots; (right) summary figure. (**B**) (left) the frequency of GATA3-expressing cells among splenic Treg cells (KJ1-26^+^CD4^+^Foxp3^+^) was plotted; (right) summary figure. The mean values ± SD (*n* = 4 per group in the experiment; one-way ANOVA; *** *p* < 0.001). One representative experiment of two experiments is shown.

Treg cells are integral to the regulation of AD development by controlling Th2-biased immune responses [4,21]. Thus, we wondered whether in vivo NGO treatment can affect the generation of antigen-specific Th2 immune responses in DO11.10 TCR Tg Balb/c mice during OVA/Alum immunization. To address this issue, NGO was i.v. injected to DO11.10 TCR Tg Balb/c mice following OVA/Alum stimulation, and the frequency of Foxp3^+^ Treg cells among CD4^+^ T cells was assessed at three weeks after OVA/Alum challenge. Upon in vivo NGO treatment, the frequency of Treg cell populations was significantly decreased in OVA/Alum-immunized mice (Figure 2A). Previous studies have reported that lung Treg cells can co-express Foxp3 and the Th2 transcription factor GATA3 in the OVA-induced airway inflammation model [12], and the adoptive transfer of Th2-type Treg cells purified from OVA-sensitized mice significantly aggravates the clinical signs of OVA-induced allergic asthma [22]. Thus, we analyzed whether NGO treatment affects the polarization of Treg cells during OVA/Alum immunization. We found that NGO significantly prevents Treg cells from acquiring a Th2-like phenotype following the injection of OVA/Alum (Figure 2B). Collectively, these results strongly indicate that NGO can control the expansion of GATA3-expressing, Th2-type Treg cells under in vivo Th2-dominant immune conditions.

### 3.3. NGO Suppresses OVA-Induced Skin Inflammation in DO11.10 TCR Tg Balb/c Mice

**Figure 3:**
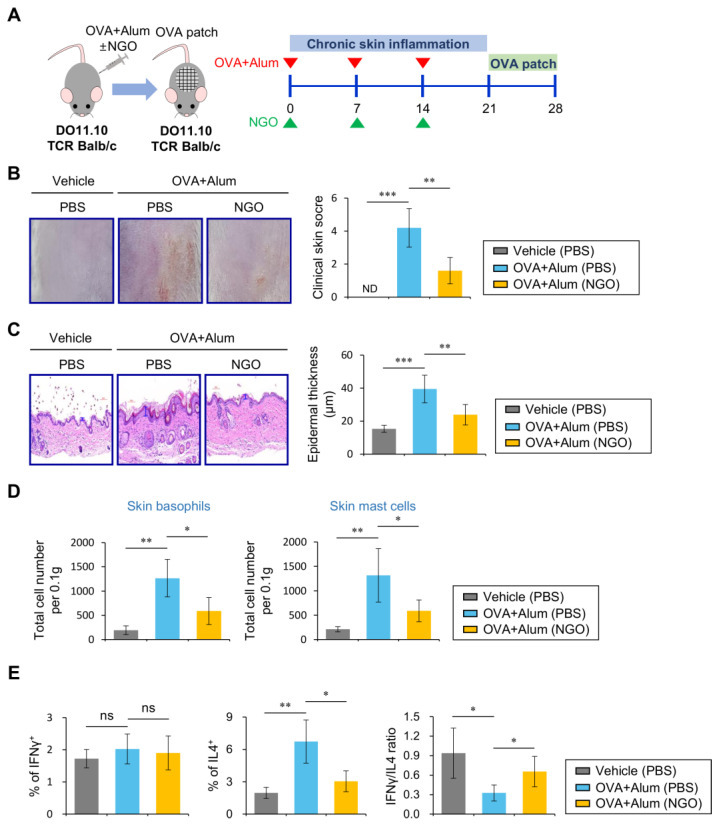
NGO injection suppresses OVA-induced skin inflammation in DO11.10 TCR Tg Balb/c mice. (**A**) DO11.10 TCR Tg Balb/c mice were immunized i.p. with Alum (1 mg) plus OVA_323–339_ peptide (100 µg) after i.v. injection of either NGO (50 µg) or PBS once a week for 3 weeks. One week after the last injection (at 21 days after immunization), mice were exposed to an OVA patch for 1 week. (**B**) The clinical symptoms were measured after patch removal (at 28 days after immunization). (**C**) Skins were prepared from these mice after patch removal. Skin lesions were sectioned and stained with H&E. The epidermal thickness was measured in 10 random high-power fields (400×) per sampled lesion. (**D**) The numbers of skin basophils and mast cells from each group were determined by flow cytometry. (**E**) The intracellular expression of IFNγ and IL4 in splenic KJ1-26^+^CD4^+^ T cells from each group was analyzed by flow cytometry. The mean values ± SD (*n* = 4 per group in the experiment; one-way ANOVA; * *p* < 0.05, ** *p* < 0.01, *** *p* < 0.001). One representative experiment of two experiments is shown. ns, not significant.

An epicutaneous challenge with OVA antigen induces the development of an AD-like phenotype dominated by Th2 responses in OVA/Alum-sensitized mice [23]. Since our findings showed that NGO suppresses Th2 responses elicited by OVA/Alum sensitization, we evaluated whether in vivo NGO treatment can protect mice against OVA-induced skin inflammation. To address this issue, we employed an OVA-induced skin inflammation model. DO11.10 TCR Tg Balb/c mice were OVA/Alum-sensitized concomitantly with i.v. injection with NGO, and seven days later, skin inflammation was induced by epicutaneous exposure to OVA using OVA-containing skin patches (Figure 3A). We measured the clinical disease scores after exposure to the OVA patch. NGO-injected mice exhibited significantly lower skin inflammation levels (particularly dryness, erythema, and edema) than PBS-injected mice (Figure 3B). Consistent with the enhanced clinical disease scores, NGO-injected mice displayed decreased epidermal thickness compared with PBS-injected mice (Figure 3C). Additionally, the number of basophils and mast cells infiltrating the skin lesions decreased in NGO-injected mice compared with the controls (Figure 3D). NGO injection into OVA/Alum-immunized mice caused a decrease in Th2 cells but not Th1 cells, ultimately resulting in a significantly increased Th1/Th2 ratio among splenic CD4^+^ T cells (Figure 3E). Together, our results strongly indicate that the NGO-mediated attenuation of Th2 responses confers a reduction in AD-like skin inflammatory pathogenesis.

## 4. Discussion

Here, we showed that in vivo NGO treatment induces a decrease in the antigen-specific Th2 population and GATA3-expressing Treg cells in Balb/c mice during OVA/Alum immunization, consequently suppressing the outcome of OVA-induced skin inflammation.

Increased IL1β production during skin inflammation reduces the expression of filaggrin, a protein essential for skin barrier function, which suggests that the down-regulation of filaggrin might be related to increased transepidermal water loss and increased permeability to allergens [6]. In addition, IL1β is known to be a critical contributor to the induction of house dust mite-specific Th2 allergic responses [7]. Previous studies have reported that small-sized and single-layer thin GO can suppress the production of IL1β and IL6 by macrophages in response to lipopolysaccharide (LPS) stimulation [17] and that GO treatment induces reduced levels of low-molecular-weight protein (LMP)7, a subunit of the immunoproteasome that is necessary for MHC I antigen presentation by DCs to CD8^+^ T cells [24]. Furthermore, NGO treatment significantly suppresses the expression of CD83, a DC maturation marker, on the surface of DCs in vitro [25]. Consistent with these previous findings, our results demonstrate that NGO injection reduces IL1β production by DCs during OVA-induced AD-like skin inflammation (Figure S1), prompting us to speculate that DCs might be one of the target cells of NGO action. Thus, the NGO-mediated reduction in DC-derived IL1β production may contribute to modulating Th2 cell induction, ultimately attenuating Th2-related, OVA-induced skin inflammation.

A selective decrease in cell number and impairment in IFNγ production in circulating natural killer (NK) cells correlates with increased risk of AD, consistent with the finding of increased sensitivity of NK cells from AD patients to apoptosis [26]. In addition, skin-infiltrating NK cells have been reported to improve the symptoms of dermatitis induced by the synthetic vitamin D3 derivative MC903 by inhibiting Th2 immune responses [27]. Emerging findings suggest that enhancing NK cell number and potency is a promising therapeutic strategy to treat AD [28]. One previous study reported that GO-based nanoclusters of anti-human CD16 (Fcγ-RIII) antibodies enhance NK cell effector functions and stimulate these cells to produce large amounts of IFNγ [29]. Thus, it will be interesting to examine whether the conjugation of NGO with anti-CD16 antibodies effectively suppresses Th2-mediated skin inflammation by increasing NK cell-derived IFNγ but decreasing Th2 cell-derived IL4.

Apart from their regulatory roles in skin immunity [30], Treg cells maintain gut homeostasis during FA by suppressing mast cell activation [31]. The absence of Foxp3^+^ T cells due to a mutation in the Foxp3 gene correlates with the prevalence of FA in humans [32]. Furthermore, innate lymphoid cell (ILC)2- and mast cell-derived IL4 contribute to allergic inflammation in the gut by suppressing the allergen-specific Treg population [33]. However, re-programming Tregs into Th2-like cells via IL4 receptor (IL4R) signaling leads to a breakdown in Treg-mediated oral tolerance and increases susceptibility to FA [11]. Thus, these results indicate that FA development can be attributed to both the loss of Foxp3 expression and conversion to Th2-like cells in Treg cells. Since NGO application constrains quantitative and qualitative alterations in Treg cells, such as a decreased Foxp3 and increased GATA3 expression during skin inflammation, it would be worthwhile to investigate whether NGO can regulate the progression of FA.

Moreover, it has been reported that the frequency of Th2-type Tregs was significantly increased, whereas the frequency of Th1-type Tregs was significantly decreased, among the PBMC of AD patients compared with healthy controls [34]. Treatment with Th2-type cytokines (i.e., IL4 and IL13) significantly decreased the gene expression of filaggrin, the main skin barrier component, in keratinocytes [35]. However, it is not fully understood whether skin Treg-derived IL4 affects Th2 immune responses and filaggrin expression in the skin microenvironment, consequently resulting in aggravated AD. Thus, it will be interesting to further investigate the function of Th2-type Tregs in AD and the effects of NGO treatment on these cellular mechanisms.

Despite the excellent potential of NGOs as therapeutic tools, there are many concerns about the toxic effects of graphene and graphene-family nanomaterials on various organs [36]. Although our data showed no significant changes in some toxicity markers (e.g., ALT and AST) following in vivo NGO injection (Figure S2), further development of biocompatible and safer NGOs is warranted. Should additional studies demonstrate their in vivo safety, NGOs will become an attractive option for treating allergic diseases such as AD.

## Data Availability

The data will be available from the corresponding author on reasonable request.

## Supplementary Materials

The following supporting information can be downloaded at: https://www.mdpi.com/article/10.3390/biom14080962/s1, Figure S1: NGO injection suppresses DC activation elicited by OVA/Alum immunization; Figure S2: In vivo toxicity of NGO in OVA-induced skin inflammation model.
