# Supplementary material for: Nano-Sized Graphene Oxide Attenuates Ovalbumin/Alum-Induced Skin Inflammation by Down-Regulating Th2 Immune Responses in Balb/c Mice
Source: Biomolecules. 2024 Aug 7;14(8):962. doi: 10.3390/biom14080962 (PMC11353088; doi:10.3390/biom14080962)
Supplement: Supplementary file 1 [file biomolecules-14-00962-s001.zip › biomolecules-3128551-supplementary.pdf]

Supporting documents to:

Nano-sized graphene oxide attenuates OVA/Alum-induced skin inflammation by down-regulating Th2 immune responses in Balb/c mice

This document includes:

-Supplementary Figures S1-2

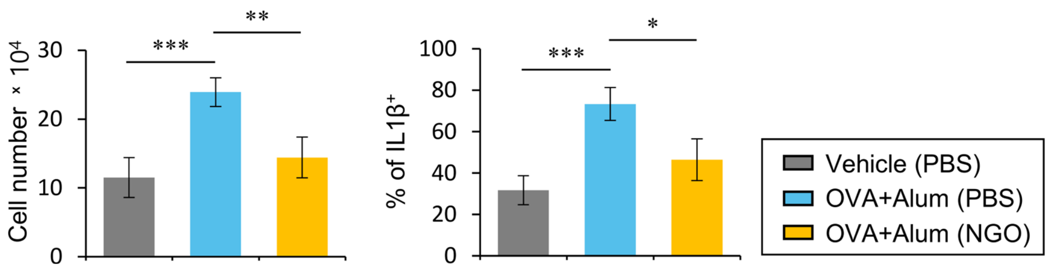

Figure S1. NGO injection suppresses DC activation elicited by OVA/Alum immunization.

4Get/DO11.10 TCR Tg Balb/c mice were immunized i.p. with Alum (1 mg) plus $OVA_{323\text{-}339}$ peptide (100 μg) after i.v. injection of either NGO (50 μg) or PBS once a week for 3 weeks. One week after the last injection (at 21 days after immunization), splenocytes were prepared from both PBS- or NGO-treated mice. (Left) The absolute cell numbers of DCs ($CD11c^+MHC\ II^+$) were assessed by flow cytometry. (Right) IL1β production by DCs ($CD11c^+MHC\ II^+$) was determined by flow cytometry. The mean values ± SD (n = 4 per group in the experiment; Student's t-test; $*p < 0.05$, $**p < 0.01$, $***p < 0.001$). One representative experiment of two experiments is shown.

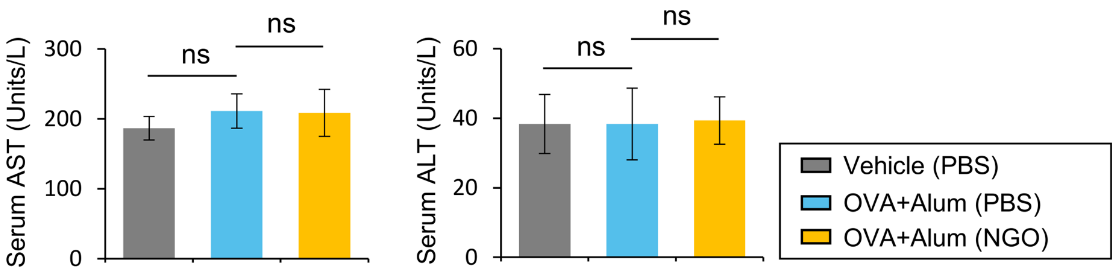

Figure S2. In vivo toxicity of NGO in OVA-induced skin inflammation model.

DO11.10 TCR Tg Balb/c mice were immunized i.p. with Alum (1 mg) plus $OVA_{323-339}$ peptide (100 µg) after i.v. injection of either NGO (50 µg) or PBS once a week for 3 weeks. One week after the last injection (at 21 days after immunization), mice were exposed to OVA patch for 1 week. Serum samples were also prepared from these mice after patch removal (at 28 days after immunization). Serum AST and ALT levels from the indicated mice were measured. The mean values ± SD (*n* = 4 per group in the experiment).
